# Addressing the need for predictive tools in postoperative abdominal wall complications after nephrectomy – Evaluation of a novel abdominal bulge grading system using computed tomography

**DOI:** 10.1177/20584601251367336

**Published:** 2025-08-26

**Authors:** Aapo Inkiläinen, Börje Ljungberg, Lennart Blomqvist, Karin Strigård

**Affiliations:** 1 Department of Diagnostics and Intervention, Urology and Andrology, 8075 Umeå University, Umeå, Sweden; 2 Department of Nuclear Medicine and Hospital Physics, 59562 Karolinska University Hospital, Stockholm, Sweden; 3 Department of Radiation Sciences, Diagnostic Radiology, 8075 Umeå University, Umeå, Sweden; 4 Department of Diagnostics and Intervention, Surgery, 8075 Umeå University, Umeå, Sweden

**Keywords:** Abdominal bulging, flank incision, abdominal wall, computed tomography, renal cell carcinoma

## Abstract

**Background:**

Abdominal bulging affects up to one-fourth of patients after flank incision, with half experiencing impaired quality of life. Identifying patients at risk for morbid bulge could improve preventive and supportive care.

**Purpose:**

To characterise muscular changes related to postoperative abdominal bulging and design a visual scoring system to grade bulge on postoperative CT scans.

**Material and Methods:**

Patients treated with open partial nephrectomy via a flank incision between 2005 and 2016 at the University Hospital of Umeå were included. Pre- and postoperative CT scans of the first 50 consecutive patients were used to characterise imaging features of the postoperative abdominal wall. From these features, a four-tiered scoring system for abdominal bulge was designed. Two independent observers tested the system on CT scans from the 50 next patients. Inter-rater reliability was assessed using Fleiss’ Kappa.

**Results:**

Common features of abdominal bulging were extracted and a four-tier visual score ranging from normal abdominal wall to severe bulge was developed. Among the patients, ∼70% had a normal abdominal wall, ∼25% had bulge score 1, ∼7% score 2, and ∼1% score 3. Inter-rater agreement was 73.5%, with Fleiss’ Kappa 0.44.

**Conclusion:**

Features of bulge were reduced muscle thickness and ipsilateral gravitational slump affecting part or all of the lateral abdominal wall. The proposed scoring system demonstrated only moderate inter-rater reliability in this pilot setting. Further research on postoperative abdominal wall changes is needed before implementing imaging-based assessments in clinical care.

## Introduction

A flank incision is used in the retroperitoneal approach to the kidney, abdominal aorta, or lumbar spine.^1–3^ Between 2015 and 2019, 51% of all patients undergoing surgery for suspected renal cell carcinoma in Sweden were treated with open surgery. Among those treated with nephron sparing surgery, 40% were performed using an open approach.^4^ The flank incision is a common open technique since it allows direct access to the retroperitoneal space.^5^ Disadvantages include division of the muscles of the lateral abdominal wall, and a risk for injury to the intercostal and subcostal nerves. These structures provide the lateral abdominal wall with motor and sensory innervation and must be actively identified and preserved during surgery.^3,6^

Abdominal bulging of the flank is due to muscular dysfunction of the lateral abdominal wall caused by nerve injury. Permanent denervation leads to muscular atrophy. A review reported the rate of bulge following a flank incision to be as high as 26%,^2^ although rates up to 56% have been observed.^7^ Some patients develop a self-limiting bulge that appears to recede within the first postoperative year, while others suffer from permanent muscular dysfunction.^8^ In a previous study, higher BMI was associated with increased frequency of bulging.^8^

In the clinical setting, a bulge is diagnosed by examining the patient in the standing and supine positions. There are currently no recommendations or guidelines regarding diagnostic imaging of bulge using computed tomography (CT) or other imaging modalities. Previous imaging studies on patients with bulge have reported 30–60 % reduced muscle thickness and increased protrusion of the abdominal wall on the flank incision side compared to the untreated side.^6,9^

It is important to differentiate between bulge and incisional hernia, as they may coexist and have different clinical implications. In an incisional hernia, there is a defect in the abdominal wall through which intra-abdominal structures may protrude. A tight hernial opening can cause strangulation of herniated tissue. The reported rate of incisional hernia after a flank incision is 5%.^8,10^

The clinical impact of a bulge varies. Around half of all patients with a postoperative bulge experience a negative effect on their quality of life, and for a few the cosmetic and functional impact is highly debilitating.^11–15^ A bulge is associated with an increased rate of chronic pain and stiffness of the abdominal wall. Functional complaints include increased fatigue, weakness of the abdominal wall, and difficulty in finding clothes that fit.^12,13^

Surgical management of abdominal bulge is difficult,^16^ and the choice of who, when, and how to treat is left to the judgment of the surgeon. As yet, no study has provided a consensus on guidelines supporting such clinical decisions.^2^

If imaging-based tools could predict severity of abdominal bulging this could contribute to the decision on when or how to perform restorative surgery. All patients operated on with open partial nephrectomy undergo preoperative radiological examination, usually CT of the thorax and abdomen. At the time of this study, follow-up CT was routinely performed at 3, 12, and 24 months postoperatively to detect any recurrent or metastatic disease. If these CT-examinations also contain information regarding the abdominal wall, especially presence, severity and predictive factors for clinical outcome of postoperative bulge, it could facilitate clinical management. Stratification of bulge severity could also lead to future research possibilities to find pre-emptive measures to avoid these injuries.

In this study, we aimed to create and, in a pilot setting, evaluate an imaging-based visual scoring system for abdominal bulge based on features identified on postoperative CT, with the intent that the score would be easy to adapt to the clinical setting.

## Materials and methods

**Figure 1.**
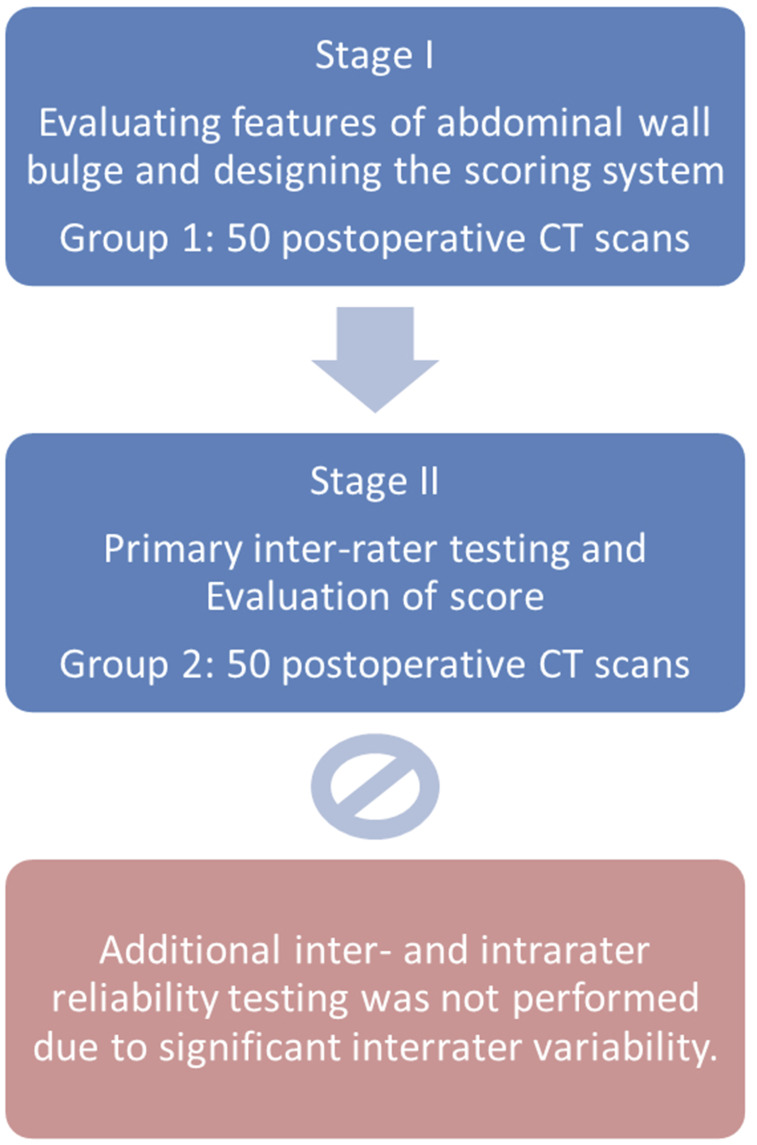
Flow chart of the study plan.

In this retrospective study, CT scans of all patients who underwent open partial nephrectomy via a flank incision at our department between 2005 and 2016 were included. Patients who did not have a postoperative CT scan were excluded. Postoperative follow-up CT scans at 3, 12, and 24 months were reviewed, focussing on the lateral abdominal wall. The standard CT protocol for routine follow-up after surgical treatment for renal cell carcinoma at our department consists of venous-phase, contrast-enhanced CT of the thorax and abdomen. Scans that deviated from this standard, such as non-contrast-enhanced scans, were not excluded. Due to the retrospective nature and the long study period, CT scans were acquired from multiple different scanner models and using varying protocols. A flow chart of the study plan is displayed in Fig 1.

### Designing the scoring system

A newly examined doctor with an interest in radiology reviewed preoperative and postoperative CT scans from routine follow-ups at 3, 12, and 24 months of the first 50 consecutive patients included, focussing on the abdominal wall.

Based on previous morphological studies^6,9^ and previous clinical experience, bulging was defined as a postoperatively occurring asymmetry or protrusion of the lateral abdominal wall with varying degrees of concurrent muscle atrophy of the abdominal wall, with no defect in any of the muscle layers.

In cases meeting the definition above, further features of the bulge were identified, and a scoring system was developed in collaboration with a board-certified radiologist.

### Evaluating and validating the score

The scoring system was evaluated in a test group of 50 CT scans from the next 50 consecutive patients. All these scans were from a 12-month postoperative follow-up. The newly examined doctor with an interest in radiology (observer (1) and a board-certified radiologist (observer (2) independently reviewed the same group of scans in sets of 10 scans at a time. After each set, individual results were compared to assess variability in scoring between the observers, taking into account a learning curve in the initial sets.

Further evaluation for inter-rater and intra-rater reliability was planned but was cancelled due to inter-rater variability between the observers in the first evaluation.

Ethics approval for this study was obtained from the Regional Ethics Committee (Dnr 2014/312-31M). Informed consent was waived due to the retrospective nature of this study.

### Statistics

STATA version 13.1 (STATACorp LP, College Station, TX, USA) was used for all calculations. The level of agreement was measured using Fleiss’ Kappa.

## Results

During the review of axial and coronal imaging planes of CT scans from the 50 first consecutive patients, we found that the commonly occurring features of bulge were on one hand reduced muscle thickness on the affected side, which could affect a part of the muscle or the entire width. On the other hand, different grades of deformity of the normal lateral abdominal wall contour were found that with increased severity led to slumping due to gravity.

**Figure 2.**
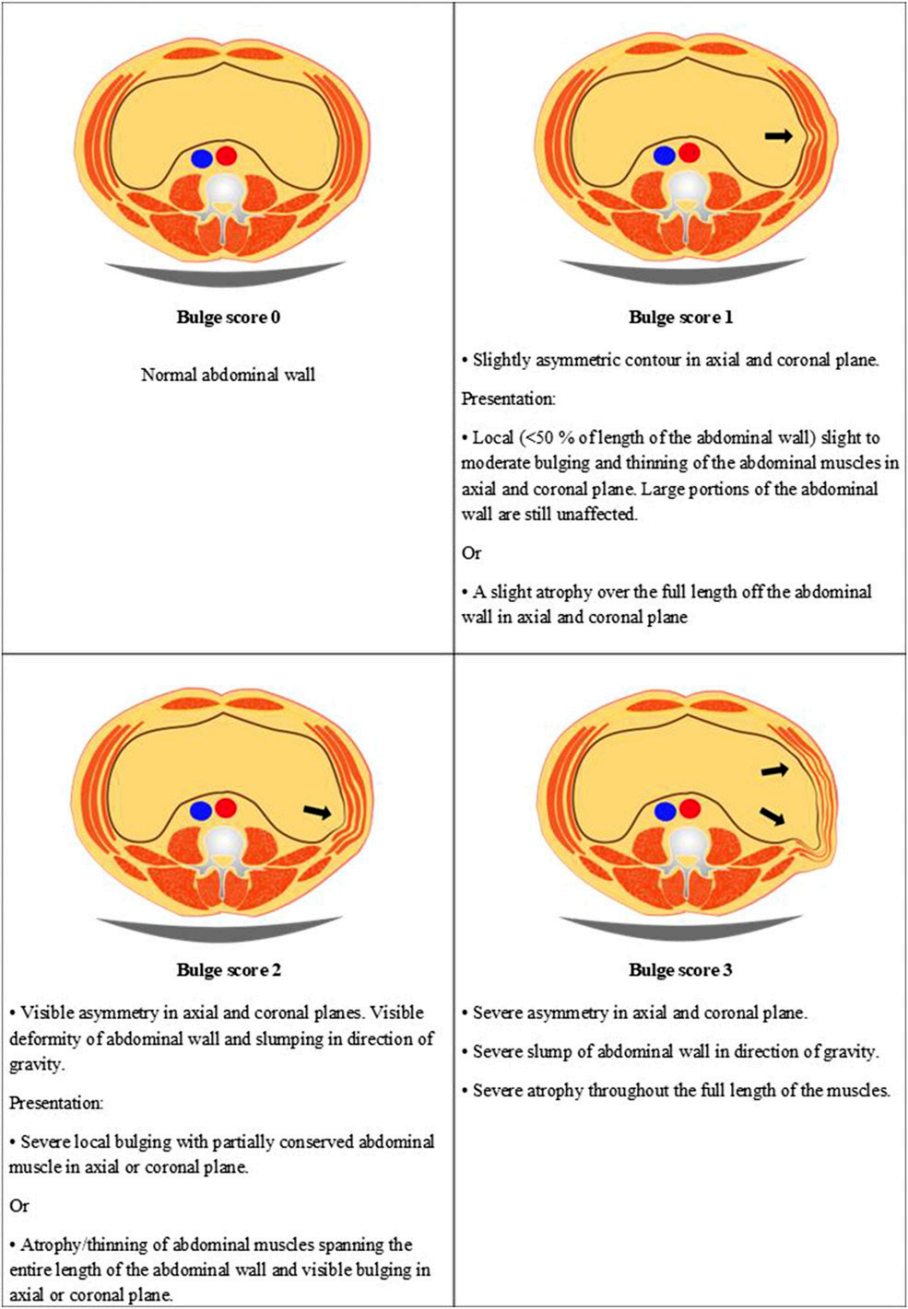
Bulge score definitions based on volumetric CT review.

**Figure 3.**
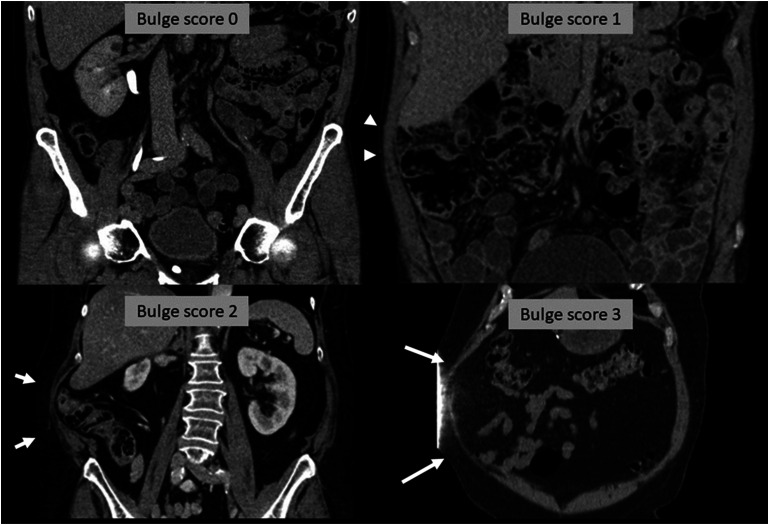
Coronal reformatted CT images showing examples of the different scores of abdominal bulge. The example for bulge score 0 was treated on the left side (right in image) while the other examples were treated on the right side (left in image). Bulge score 1 (arrowheads) with local thinning of right lateral abdominal muscles. Bulge score 2 (short arrows) with severe local bulging of the right lateral abdominal muscles. Bulge score 3 (long arrows) with severe thinning and asymmetry of the right anterolateral abdominal muscles.

Based on these bulge features, a scoring system comprising four different degrees of abdominal bulge was developed (Figs 2 and 3), ranging from a normal abdominal wall to a severe bulge.

**Figure 4.**
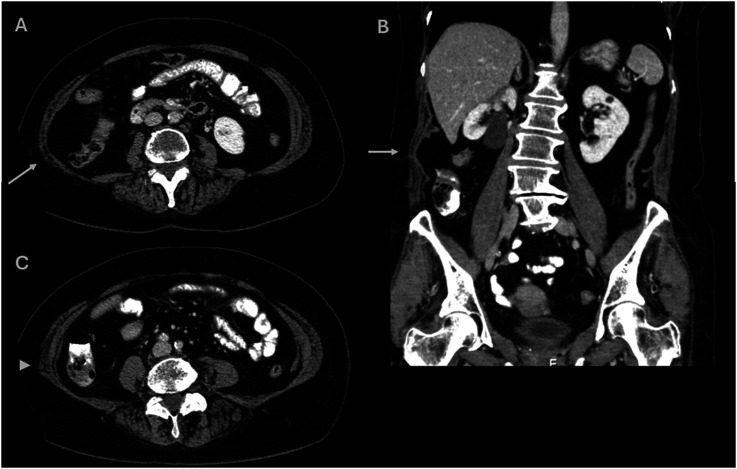
Example of bulge score 1 in axial and coronal reformatted CT images. A and (b) Moderate focal muscle atrophy (arrows) in the upper right abdominal wall, at L3 level in axial plane (a) and coronal plane (b). This was graded as bulge score 1 by both observers. (c) Axial plane at L5 level in the same patient shows only slight asymmetry (arrowhead) in muscle volume.

**Figure 5.**
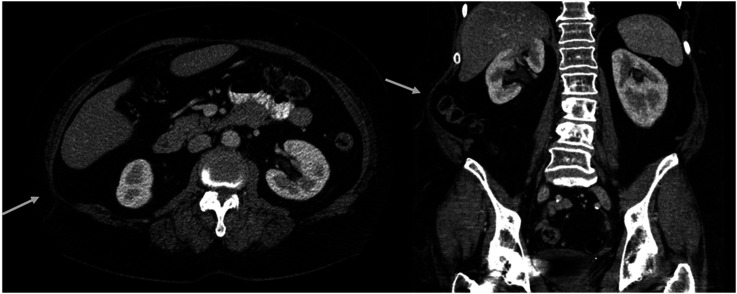
Example of bulge score 2 in axial and coronal reformatted CT images. High-grade focal atrophy in right upper posterior abdominal wall, graded as bulge score 2 by both observers.

When evaluating the bulge score observer 1 found 14 cases (28%) with abdominal wall changes considered to be bulging (bulge score 2-4), while observer 2 found 17 cases (34%) (p = .52) (Table 1). Examples of scoring for bulge score 1 and bulge score 2 are displayed in Figs 4 and 5, respectively.

**Table 1.** Abdominal wall rating by the two observers.

	Bulge score 0	Bulge score 1	Bulge score 2	Bulge score 3
Observer 1	36	10	4	0^a^
Observer 2	33	13	3	1^a^

^a^This case was judged by observer 2 to be a hernia with concomitant bulge, while observer 1 judged it to be a hernia.

Inter-rater agreement for scoring bulge between the two observers was 73.5%, with Fleiss’ Kappa of 0.44 which represents only moderate agreement. When dichotomising results into bulge or no bulge (score 0 vs scores 1-3) an agreement of 81.6%, with a Kappa 0.58 which represents moderate agreement. Analysing each category separately showed no significant agreement.

## Discussion

In this study, we found several imaging features associated with postoperative abdominal wall bulge. However, the subjective bulge score model proposed in this study was found to be associated with moderate inter-rater variability.

When defining imaging features of bulge, we found that the location and degree of maximal muscle atrophy varied. Ozel *et al*^6^ used ultrasonography to measure muscle thickness at fixed anatomical positions in patients after open donor nephrectomy via flank incision. In two patients complaining of postoperative abdominal wall bulging, they found the thicknesses of the rectus abdominis and the lateral abdominal wall muscles were decreased by 45% and 60%, respectively, on the affected side compared to the non-operated side. Ultrasound is highly user dependent and only visualizes small portions of the abdominal wall in the field of view, which makes comparison of sides difficult, and the extent of abdominal wall changes may be difficult to assess adequately. Additionally, gravitational slump of the abdominal wall might be difficult to visualize without side-by-side comparison of the whole abdominal wall.

Crouzet *et al*^9^ measured the volume of the lateral abdominal wall on CT scans using volumetric segmentation. Their software allowed semi-automated selection of tissues based on density (Hounsfield value), and manual correction of the selected volume was needed to remove accidentally included organs. Their idea of volumetric segmentation is interesting since it produces multiple quantifiable variables. Variables such as muscle volume, thickness variation, and degree of deformity, may be quantified from one segmented volume. Differences in attenuation allow for identification and characterisation of fatty atrophy. However, precise manual anatomic segmentation is time-consuming. While it is adequate on a small scale, it is impractical in large studies or in clinical practice.

There are obviously some limitations to this pilot study. Due to differences in abdominal wall shape on the scans and occasional gravitational shift in the supine position in obese patients, visual scoring proved to be difficult. The scoring system was designed based on scans from only 50 patients. This amount may be too limited to be fully representative. Bulge varies in severity and severe cases are infrequent. However, considering that the initial scoring system was based on one preoperative and three postoperative CT in each of the reviewed 50 patients, the initial evaluation of abdominal wall changes should have provided a good understanding of abdominal bulge features. In the few severe cases, the abdominal wall changes were also more pronounced making them easier to identify. It is more difficult to draw the line between a normal abdominal wall and one with discrete pathological changes, which in retrospect could have been easier to visualise if a comparison to previous radiology had been included in the score.

The proposed scoring system used descriptive terms for the four bulge scores, which allowed for more freedom for the observer but unfortunately yielded a non-consistent method. It is possible that a more rigid approach, with more specific definitions and a checkbox system, could have provided more consistent results. However, considering the heterogeneity of the normal abdominal wall, a more rigid approach would make the system too complex and defeat the goal of providing an easy-to-use clinical tool that correlates with clinical symptoms.

When imaging an abdominal wall hernia, it is common to perform CT with Valsalva’s manoeuvre. Although earlier studies have reported the importance of Valsalva’s manoeuvre for ventral abdominal wall hernias^17^ more recent studies with modern CT equipment have shown that incisional hernias and ventral abdominal wall hernias are rarely missed on a regular CT scan. Valsalva’s manoeuvre is useful for sliding type hernias such as femoral hernias where 43% were not visible on regular CT.^18^ This technique was not used in the present study. It is possible that a bulge would also become more apparent during Valsalva’s manoeuvre due to asymmetrical abdominal wall laxity following the denervation injury, similarly to rectus diastasis which becomes more apparent with the manoeuvre.^18^ This would, however, require specific imaging studies.

Visual radiological scoring systems are used in clinical practice. Another uroradiological scoring system that is widely used in clinical practice is the Bosniak classification which stratifies malignancy risk of cystic renal masses. The Bosniak classification has been associated with inter-reader variability with reported kappa values of 0.37 and 0.55.^19–21^ Like our proposed bulge score, the greatest variability has been in the middle categories, that is, when attempting to stratify Bosniak II, IIF, and III cystic renal masses. A more successful and simple visual scoring system is the five graded medial temporal atrophy scale with reported 90% agreement and a kappa value of 0.86.^22^ An advantage of this scale over the bulge score, is that grading is based on a single standardised image which allows more precise definitions for the categories.^23^ Furthermore, neuroanatomic structures do not shift with gravity to the same degree as abdominal structures, and standardised images can be constructed even if the patient’s head is tilted during examination. In contrast, the bulge scoring system required evaluation of a stack of CT images, and the abdominal contour shifts according to the patient’s position.

The subjective nature of the proposed bulge scoring system may explain the inter-reader variability in the present study. A more objective method with fully automated segmentation of the abdominal wall might be more useful if it allows for multiple quantifiable measurements that can be correlated to clinical data. Such a technique would not only be bulge-specific but could potentially be adapted to the whole spectrum of abdominal wall pathology, for example, evaluation of other abdominal wall pathology such as hernias including parastomal hernia, or even in preoperative planning of surgery.

In conclusion, features of abdominal wall bulging on postoperative CT were defined and a visual scoring system for abdominal bulge based on postoperative computed tomography was proposed. The scoring system in this pilot study was associated with only moderate inter-reader agreement and therefore impractical for clinical use in its present form. Further work to establish imaging-based techniques to grade postoperative abdominal bulge and reduce variability between readers is clearly warranted.
